# Neural Plasticity Is Involved in Physiological Sleep, Depressive Sleep Disturbances, and Antidepressant Treatments

**DOI:** 10.1155/2017/5870735

**Published:** 2017-10-18

**Authors:** Meng-Qi Zhang, Rui Li, Yi-Qun Wang, Zhi-Li Huang

**Affiliations:** Department of Pharmacology and Shanghai Key Laboratory of Bioactive Small Molecules, School of Basic Medical Sciences, State Key Laboratory of Medical Neurobiology, Institutes of Brain Science and Collaborative Innovation Center for Brain Science, Fudan University, Shanghai 200032, China

## Abstract

Depression, which is characterized by a pervasive and persistent low mood and anhedonia, greatly impacts patients, their families, and society. The associated and recurring sleep disturbances further reduce patient's quality of life. However, therapeutic sleep deprivation has been regarded as a rapid and robust antidepressant treatment for several decades, which suggests a complicated role of sleep in development of depression. Changes in neural plasticity are observed during physiological sleep, therapeutic sleep deprivation, and depression. This correlation might help us to understand better the mechanism underlying development of depression and the role of sleep. In this review, we first introduce the structure of sleep and the facilitated neural plasticity caused by physiological sleep. Then, we introduce sleep disturbances and changes in plasticity in patients with depression. Finally, the effects and mechanisms of antidepressants and therapeutic sleep deprivation on neural plasticity are discussed.

## 1. Introduction

Depression, which is characterized by a pervasive and persistent low mood and anhedonia, greatly impacts patients, their families, and society. It contributes largely to the global disease burden [1] and is associated with increased risks of several other diseases, which can further increase the economic burdens of individuals [2, 3]. In clinical practice, sleep disturbances are among the common complaints of depressed patients and negatively affect the quality of their lives. Studies demonstrated that sleep can facilitate neural plasticity, and changes in plasticity have been observed in depressed patients. However, therapeutic sleep deprivation exerts a rapid and robust antidepressant effect in patients with broadly defined depression. These facts raise the possibility that depression and accompanying sleep disturbances share a common origin. In other words, they may represent different phenotypes of the same pathophysiological process. To address this question, we first examine the macro- and microstructures of sleep and present evidence of how sleep facilitates neural plasticity. Then, we list the sleep disturbances and changes in neural plasticity in depression, including studies on humans and animals, and explain the common mechanisms. Next, we analyze the effects of antidepressants on neural plasticity and their mechanisms. Finally, we consider sleep deprivation as a therapy for depression and explain the consequences and mechanism in detail.

## 2. Sleep and Neural Plasticity

### 2.1. The Overall Structure of Sleep

Sleep or sleep-like state is ubiquitous to most living organisms. While awareness of the surroundings seems to be deliberately lowered or even blocked during the deepest stage of sleep, many processes continue to function. In terms of characteristics of the electroencephalogram (EEG), sleep in mammals can be divided into two distinct stages: rapid eye movement (REM) sleep and non-REM (NREM) sleep. NREM sleep in humans can be categorized further into 3 stages: stage 1 (N1), stage 2 (N2), and stage 3 (N3) [4]. N1 represents the transition from wake to sleep since predominant EEG activities shift from 14 to 30 Hz in wakefulness or 8–12 Hz in quiet rest to 4–7 Hz oscillations [5], while *κ*-complex events and sleep spindles occur in N2. *κ*-Complexes protect sleep from outside interference [6] and facilitate generation of sleep spindles [7], which usually last no more than 2 s and range from 11 to 15 Hz [8]. N3, which is a deeper NREM sleep stage compared with N1 and N2, is dominated by slow wave activity (SWA) ranging from 0.5 to 4 Hz that includes neocortical slow oscillations ranging generally from 0.5 to 1 Hz [5]. In addition, a special type of oscillation, known as sharp wave ripple (SWR) complexes, can be observed at the level of the hippocampus mainly during N3 [9, 10]. These SWR complexes, which range from 100 to 250 Hz, consist of sharp waves that originated in the CA3 region of the hippocampus and produce fast ripples in the CA1 region. In contrast, REM sleep is dominated by theta activity ranging from 4 to 8 Hz [11] and is associated with persistent muscle atonia and bursts of eye movement.

### 2.2. Generation of Different EEG Characteristics in Sleep

Our knowledge of intrinsic networks underlying different EEG activities grows as methodologies develop. For instance, SWA is a consequence of autonomous neocortical slow oscillations that result from interactions between excitatory and inhibitory neurons in the cortex [12]. Intracellular and extracellular recordings have demonstrated that the slow oscillation, which consists of up and down states, enters up state if an inside or outside signal stimulating the local cortical network is strong enough to counter local inhibition [13, 14] and the local network has passed its refractory period [14–16]. This local excitation spreads as positive feedback and leads to the synchronization visible in the EEG [12]. The thalamocortical neurons, which *in vitro* show strong intrinsic rhythms similar to the up and down states [8, 17, 18], are reciprocally connected with the cortex and depolarize in advance of the up states [14, 19–21]. Recent studies using optogenetics revealed that selective activation of thalamocortical neurons can induce the up state in the slow oscillation [22] and SWA [23]. These findings indicate that the thalamus is crucial in generating SWA [18] and implicate the thalamocortical network as an inseparable structure in regulating SWA [24]. Sleep spindles are generated by an interaction between thalamocortical relay cells and GABAergic neurons in the thalamic reticular nucleus [25–27]. Generation of theta activity, which is usually recorded at the hippocampus, involves the projection from the brainstem containing the center responsible for REM sleep [28] to the medial septum (MS) via the hypothalamus [29]. Pacemaker cells in the MS, which spontaneously fire in the valley of theta activity [30], provide inhibitory input to CA1 pyramidal cells [29]. The hippocampus also sends feedback to the medial and lateral septum [31], which synchronizes between the 2 structures. Conversely, the entorhinal cortex (EC) excites the hippocampus with cortical information via its direct glutamatergic projections to the CA1, CA3, and dentate gyrus [32, 33]. Recent studies showed that only the medial EC (MEC) appears related to generation of theta activity [34] and is also under control of GABAergic neurons in the MS [35]. Within the hippocampus, the oscillatory activation of the EC transmitted by the perforant path generates prominent theta activity in the dentate gyrus and then excites the CA3 and CA1 regions to compete with oscillatory inhibition driven by the MS. Furthermore, several other brain regions, such as the dorsal raphe nucleus (DRN), are also involved in control of theta generation through connections with the septal complex, which is composed of the MS as well as the vertical and horizontal limbs of the diagonal band of Broca [36]. In addition, pyramidal cells and interneurons in the medial prefrontal cortex (mPFC) can be excited by CA1 pyramidal cells from the ventral part of the hippocampus [37], and the amygdala complex, which is a critical interface for emotional responses, is reciprocally connected with the regions that are implicated in theta generation.

### 2.3. Implications of EEG Changes in Neural Plasticity

While the mechanisms of specific EEG activities have been elucidated, we still lack a universal theory to answer the mysterious question of why we sleep. One intriguing possibility is that sleep is needed because of neural plasticity, which is a process that fundamentally decides how we interact with the world [38, 39]. Neural plasticity is an umbrella term that may refer to structural alterations in the brain on a large scale, such as cortical remapping and changes in total weight, or on a microscopic scale, such as changes in size and density of neurons and glia. At the single cell level, synaptic plasticity describes the changes in strength of existing synapses, in synapse number or size, or in morphological structures that contain synapses [40].

The first line of evidence supporting a relationship between sleep and neural plasticity comes from patients suffering from insomnia. They exhibit reduced gray matter in subregions of the prefrontal cortex (PFC) [41, 42] and a smaller hippocampal volume [43, 44]. In addition, patients with primary insomnia show decreased sleep-dependent memory consolidation, which is commonly considered an indicator for neural plasticity, in procedural and declarative learning [45, 46].

Deeper examination of EEG studies, which directly and accurately reflect collective changes in the brain, reveals a profound link between sleep and neural plasticity. SWA is recognized as a measure of sleep need [47]. It increases with the prolongation of wakefulness and decreases gradually during sleep [48]. The increase of SWA during sleep has been shown to be directly associated with long-term potentiation (LTP) rather than prolonged wakefulness, since areas with increased LTP exhibit enhanced SWA while a reduction in LTP-related molecules blunts the SWA peak [49, 50]. Several studies have demonstrated that enhanced SWA is spatially and temporally associated with LTP during wakefulness [51, 52]. Computational studies indicate a relationship between stronger synaptic connections and higher SWA [53, 54]. Furthermore, studies found that approximately 5% of gene transcription in the rat cortex is under control of the sleep-wake cycle [55]. In particular, mRNA levels of genes associated with building new synapses and strengthening existing synapses increase in both cortical and hippocampal [55]. In addition, adenosine, which is closely associated with homeostatic regulation of sleep [56, 57], has been reported to impact neural plasticity via adenosine A_1_ receptors (A_1_Rs) [58]. This is especially true in the hippocampus where extracellular levels of adenosine increase [59], and these increases colocalize with A_1_Rs [60]. When the increase of extracellular levels of adenosine is attenuated, hippocampal LTP, which is low after sleep deprivation returns to normal. The same effect is observed when 8-cyclopentyl-1,3-dimethylxanthine, an A_1_R antagonist, is chronically infused into the brain, which suggests that adenosine may play a role in regulation of hippocampal plasticity [61–63].

These lines of evidence give rise to the synaptic homeostasis hypothesis (SHY), which was developed by Tononi and Cirelli [64–66]. The main claims of the SHY are as follows: (1) Wakefulness is related to synaptic potentiation and increases in synaptic weight. (2) The amount of SWA during sleep adjusts according to the level of synaptic potentiation during preceding wakefulness in a spatiotemporal manner. (3) The increased SWA represents a generalized depression, namely downscaling [64–66]. This third claim is supported by reduced expression of synaptic markers [67, 68] and a net elimination of dendritic spines [69–71] during sleep. Indeed, when animals are placed in an enriched environment before sleep, expression of the immediate early gene, zif-268, is enhanced in REM and NREM sleep [72]. However, in comparison with activity-dependent synaptic scaling, this downscaling process should only affect recently potentiated synapses [65], which is conceptually different from long-term depression. A recent convincing study by de Vivo [73] using three-dimensional electron microscopy showed that the axon-spine interface (ASI) decreased by approximately 18% after sleep compared with during wakefulness. The animals were divided into 3 groups: (1) the spontaneous wake group in which brain tissues were obtained at 03:00, (2) the enforced wake group that was exposed to novel objects during day in which brain tissues were obtained at 15:00, and (3) the spontaneous sleep group in which brain tissues were obtained at 15:00. The ASI of animals in the spontaneous sleep group exhibited a significant reduction compared with the ASI of animals in the spontaneous wake and enforced wake groups, and the reduction was proportional to ASI size. This evidence is considered solid proof of the third claim in the SHY. Although some studies using sleep deprivation failed to find changes in markers of neuronal degeneration, stress, or apoptosis [74–76], there is certainly a mutual relationship between sleep and neural plasticity. However, a more elegant explanation is required to form a universal theory.

## 3. Mutual Mechanisms Underlying Sleep Disturbances and Neural Plasticity Anomalies within Depression

### 3.1. Sleep Disturbances and Neural Plasticity Anomalies within Depression

Depression is strongly associated with sleep disturbances [77]. Sleep disturbances are common complaints of patients suffering from depression, ranging from problems with falling asleep, frequent nocturnal awakenings, early morning awakenings, or a disturbed sleep duration [78, 79]. In turn, an epidemiological study showed that compared with persons free from sleep problems, individuals with insomnia are more likely to develop depression. The persistence of insomnia is associated with progress of new depressive episodes [80, 81].

Sleep EEG recordings provide more details on anomalies in sleep architecture. Delays in sleep onset, decreases in REM latency, and increases in REM sleep amounts along with sleep fragmentation are observed [82]. The cost of an increase in REM sleep is a reduction in NREM sleep, especially N3 [78]. Moreover, as an indicator of NREM sleep intensity, SWA should be highest in the first sleep cycle, and this is the case in the control subjects. However, in depressed patients, SWA is higher in subsequent sleep cycles [83], which suggests a suppressed generation of SWA.

In line with these findings, other studies indicate that depression is associated with changes in neural plasticity. The most concordant one is the observed decreased volume of the PFC and hippocampus [84–87]. Studies using rodent models revealed that stress can lead to atrophy and loss of neurons and glia in the PFC and hippocampus [88, 89], which is consistent with a decrease in synapse number in the PFC of patients with depression as demonstrated in postmortem studies [90]. In addition, repeated restraint stress induces a decrease in number and length of apical dendrites and spine synapses in pyramidal neurons of the mPFC [91]. Sleep fragmentation, which is a common sleep problem in depressed patients, causes a loss of N-methyl-d-aspartate (NMDA) receptor-dependent LTP in the hippocampal CA1 region [92]. Similarly, electrophysiological and immunoblotting studies indicate that insufficient sleep can impair LTP and facilitate LTD in the hippocampal CA1 area of mice, which is associated with selective augmentation of the number of NMDA receptor NR2A subunits and an increase in the NR1A/NR2B ratio [93, 94].

Recent studies suggest that the infralimbic PFC, which is responsible for processing emotional information, regulates the ventral tegmental areas (VTA) via the amygdala and ventral subiculum [95]. Thus, impaired functional connectivity of this circuit may lead to improper responses to rewards and anhedonia [96]. The ventral striatum is particularly crucial in coding and updating predictions about a reward based on previous experience, while the dorsal striatum is involved in defective action-reward contingency learning [97]. Therefore, it is not surprising to find aberrant activity in these 2 areas in depressed patients [98]. Interestingly, a recent study conducted by Oishi et al. using chemogenetics demonstrated that activation of VTA dopaminergic neurons induced a robust increase in wakefulness [99]. In contrast, the ventral striatum nucleus accumbens (NAc), which plays a key role in reward functions, has been found to increase sleep via dopamine D_2_ receptors [100]. Moreover, the amygdala complex is known to regulate REM sleep based on reciprocal connections with ventrolateral periaqueductal gray (vlPAG) in the midbrain and the lateral pontine tegmentum (LPT) and sublaterodorsal nucleus (SLD) in the brainstem [101–103]. This overlap in neural circuitry of depression and sleep regulation may shed light on the mutual mechanisms that account for genesis of depression, depressive sleep disturbance, and neural plasticity [58].

### 3.2. Mutual Underlying Mechanisms

Depression is classified as a neurochemical disorder and has long been considered a mood disorder in which stress plays a vital role via an impaired monoaminergic neurotransmitter, usually serotonin (5-HT) [104–106]. The serotonergic system in the brain is located at the DRN and median raphe nucleus (MRN). These 2 nuclei project to many wake-promoting brain regions such as the basal forebrain, thalamus, hypothalamus, and cortex [107]. In addition, the extended amygdala and PFC are also innervated by the DRN and MRN [107]. Recent studies utilizing optogenetics found that activation of 5-HT neurons induced an increase in wakefulness and sleep fragmentation [108] partially due to corelease of glutamate [109]. Moreover, the DRN and MRN inhibit the SLD during NREM sleep and wakefulness, while this inhibition withdraws during REM sleep and gives rise to the glutamatergic projection in the SLD to generate muscle atonia [110, 111]. In addition, the decreased inhibitory inputs from the DRN and MRN also disinhibit the pedunculopontine and laterodorsal tegmental nuclei (PPT/LDT) [28] and result in generation of theta activity via the ascending pathway targeting the MS [29]. 5-HT also participates in tuning the balance between excitation and inhibition [112]. In the brain, 16 types of 5-HT receptors have been identified [113], and the metabotropic 5-HT_1A_ receptors (5-HT_1A_Rs) are the dominant type in the PFC [114]. The layer 5 pyramidal neurons (L5PyNs) of the PFC express 5-HT_1A_Rs in both soma, initial parts of axons and dendrites [114–116]. Moreau et al. showed that 5-HT_1A_Rs in L5PyNs play an important role in controlling output signals of the PFC. Although most postsynaptic 5-HT_1A_Rs are expressed in glutamatergic neurons in the PFC, GABAergic neurons also express 5-HT_1A_Rs and project onto the dendrites of pyramidal cells [114]. This appears to explain the anomaly of SWA in depression, which may be due to an imbalance of 5-HT_1A_R modulation of excitation and inhibition [117, 118].

Dopamine is another monoaminergic neurotransmitter that has attracted much attention. As the last fully developed monoaminergic system in the brain [119], the dopamine system plays roles in many brain functions including locomotion, reward, motivation, learning, and cognition [120]. Although 5-HT is traditionally linked with the pathophysiology of depression, it may not account for other key characteristics of depression, such as anhedonia and amotivation [121], whereas dysfunction in the dopamine system is consistent with these characteristics [122]. Excessive physiological or emotional stress and subsequent anxiety can give rise to major psychiatric disorders such as depression [123]. When subjects are exposed to transient stressors, dopaminergic neurons in the medial VTA exhibit short-term inhibition [124]. However, following exposure to a prolonged stressor, activity of dopaminergic neurons in the medial-lateral VTA increases briefly before a prolonged suppression, and the level of dopamine in the PFC and NAc increases [125, 126]. Abnormal neuronal activity of the dopaminergic system can be normalized by inhibiting the hippocampus, and decreased responsivity of the dopaminergic system is driven by the amygdala [127, 128]. Further investigations revealed that in animal depression models utilizing stress factors, the hyperactive infralimbic PFC activates the amygdala, which then suppresses the VTA, especially the medial part, through GABAergic neurons in the ventral pallidum and reduces normal reward-related activity in this brain region [95, 129, 130]. In addition, stressors, such as forced swimming, increase the alpha-amino-3-hydroxy-5-methyl-4-isoxazolepropionic acid (AMPA)/NMDA ratio of excitatory synapses on VTA dopaminergic neurons [131, 132]. This process of potentiation is initiated as soon as 2 h after stressor delivery and maintained at least 1 d [132], and blockade of both AMPA and NMDA receptors in the VTA can prevent increased dopamine levels in the PFC [133]. However, the reaction of VTA dopaminergic neurons to stress differs based on the projection site of these neurons. An increased firing rate is only found in neurons projecting to the NAc [134–137], while those projecting to the PFC decrease firing rate in the social defeat animals [138]. Nonetheless, when firing rate is restored to normal, social interaction behaviors also normalize [135, 136, 138]. A study by Tye et al. [129] using optogenetics to stimulate VTA dopaminergic neurons while blocking the dopamine receptors in the NAc failed to reverse depressive behaviors induced using a chronic mild stress model, which suggests an essential role of the VTA-NAc circuit in stress- and depression-related behaviors such as anhedonia [139]. Interestingly, the same VTA-NAc circuit also plays an important role in the mesolimbic dopamine system in regulating the sleep-wake cycle as mentioned above. Substantial evidence has shown that the VTA promotes wakefulness by modulating the NAc and receives glutamatergic, GABAergic, serotoninergic, and cholinergic modulation from other brain regions such as the LDT, PAG, and DRN [100]. Sleep-wake regulation of the NAc is under control of the PFC, ventral hippocampus, VTA, thalamus, and amygdala and is achieved by traditional direct/indirect pathways of the basal ganglia [100].

Adenosine, as an endogenous sleep promoter, is also involved in neural plasticity, and activation of A_1_Rs suppresses LTP [140]. Zgombick et al. [141] proposed that since A_1_Rs and 5-HT_1A_Rs are colocalized and share G proteins in several brain regions, they may affect intracellular signaling cascades together. These effects are mediated by the cyclic adenosine monophosphate (cAMP) signaling pathway since A_1_Rs are inhibitory G protein-coupled receptors. The cAMP-response element binding protein (CREB), which can be activated by the cAMP-protein kinase A (PKA) signaling pathway [9, 142], is vital to long-lasting hippocampal synaptic plasticity [143, 144]. In addition, expression of brain-derived neurotrophic factor (BDNF), which is a critical promoter of neurogenesis, neuronal survival and synaptic plasticity [145, 146], is under control of CREB [147]. BDNF decreases in expression and function in the PFC and hippocampus in animal models, which is crucial in the genesis of depression, as well as in the blood of patients with depression [148–150]. Blockade of BDNF release causes atrophy of neurons in the hippocampus [151] and mPFC [152] in mice, while heterozygous deletion of BDNF reduces spine density and dendrite of neurons in the hippocampus and PFC along with a decreased volume of the hippocampus [153, 154]. Recent research suggests that adenosine A_2A_ receptors (A_2A_Rs) are arguably more important than A_1_Rs in homeostatic regulation of sleep [56]. However, little is known about their role in neural plasticity. Our work has demonstrated that increased REM sleep induced by bilateral olfactory bulbectomy is associated with A_2A_Rs in the olfactory bulb and can be normalized by acute administration of fluoxetine, but depressive behaviors remain the same [155, 156]. While the REM-suppressing role of A_2A_Rs in the olfactory bulb can be explained by mutual connections with REM-regulating nuclei in the brainstem via the piriform cortex and amygdala, depressive behaviors induced by bilateral olfactory bulbectomy seem to be long-lasting and need further investigation. Answering this question should increase our understanding of how adenosine regulates neural plasticity.

The largely overlapping mechanisms of sleep regulation and genesis of depression suggest that they may share common mechanisms, one of which, as we suggest in the current review, may be neural plasticity. Sleep dysfunction impairs neural plasticity and vice versa. Human patients who suffer from depression, as well as animal depression models, show changes in neural plasticity. However, it is unlikely that sleep disturbances lead to genesis of depression, because many other neurological disorders also involve sleep disturbance. In our point of view, genesis of depression changes neural function in regions of the brain that are important for sleep regulation. This then leads to sleep disturbances, which reduces sleep quality and further facilitates depression.

## 4. Neural Plasticity Involved in Antidepressant Treatment

### 4.1. Typical Antidepressants Restore Neural Plasticity

Despite the high rate of resistance and notably long delay before taking effect, typical monoamine-based antidepressants are still the first choice in treatment of depressed patients since they were discovered fortuitously more than 50 years ago [157]. Their appearance provided a possible interpretation of the biological basis of depression and guided development of a series of more specific medications in the following decades, including tricyclic antidepressants (TCAs), monoamine-oxidase inhibitors (MAOIs), selective norepinephrine reuptake inhibitors (NARIs), selective 5-HT reuptake inhibitors (SSRIs), 5-HT/NE reuptake inhibitors (SNRIs), and 5-HT2 receptor antagonist/reuptake inhibitors. However, the discrepancy between acute changes of extrasynaptic monoamine levels and their delayed onset of action implicates other more direct and rapid changes in addition to the altered monoamine neurotransmitter system in the neurobiological basis of depression.

Growing evidence indicates that chronic treatment with antidepressants enhances neural plasticity at both cellular and functional levels. Chronic treatment with the SSRI, fluoxetine, enhances LTP and synaptic transmission in the dentate gyrus of the hippocampus, upregulates dendritic spine density in the cerebral cortex and hippocampal CA1 and CA3 fields, and blocks atrophy of dendrites and spines caused by chronic stress exposure [158–160]. It also restores neuronal plasticity in the adult visual system of rats [161]. The change in synaptic plasticity may act through local BDNF and contribute to extinction of conditioned fear by remodeling memory circuitry [162]. Administration of fluoxetine and imipramine has been reported to remodel dendritic and synaptic contacts in the hippocampus and PFC after chronic stress exposure [163]. In addition, evidence suggests that treatment with tianeptine overcomes blocking of LTP induction caused by inescapable stress [164]. Moreover, amitriptyline and mianserin have been reported to reverse bulbectomy-induced reduction in dendritic spine density in the hippocampus [165]. These studies implicate an important role of neural plasticity in antidepressant effects of these conventional medications.

### 4.2. Mechanisms Underlying Changed Neural Plasticity

#### 4.2.1. BDNF

BDNF is thought to play a pivotal role in the pathophysiology of depression and the neuroprotective effects of conventional antidepressants. It has been shown clearly that stress and glucocorticoids downregulate the expression of neurotrophins including BDNF and their receptors in the hippocampus [166, 167]. Postmortem studies also showed a decrease of BDNF protein and mRNA expression in the hippocampus of depressed suicide patients [168, 169], and this decrease can be reversed after chronic treatment with many different classes of antidepressants, including MAOIs, NARIs, SSRIs, and some atypical antidepressants [170, 171]. Furthermore, reduction of serum levels of BDNF in depressed patients can be partially normalized after administration of antidepressants [172, 173].

It is expected that BDNF can affect neural plasticity. Haploinsufficient BDNF mice have shorter and simplified CA3 dendrite spines [153]. Mice with a human loss-of-function BDNF gene variant, Val66Met, exhibit an impaired synaptogenesis in the PFC [152] and more prominent changes in dendritic spine density in the PFC and amygdala after stress [174]. In addition, their anxiety-related behaviors are increased and cannot be normalized by treatment with the antidepressant fluoxetine [174, 175]. Volunteers with the Val66Met polymorphism are more vulnerable to depressive symptoms if they are exposed to early-life stress [176]. Furthermore, heterozygous BDNF knockout mice show a blunted antidepressant effect of imipramine in the forced swim test [177]. Taken together, these studies support BDNF involvement in antidepressant effects and modulation of neural plasticity by conventional antidepressants.

#### 4.2.2. Neuroplasticity-Related Signaling Pathways

The delayed action of typical antidepressant treatments suggests a role of receptor-coupled signal transduction proteins and their genes. Stress and depression disrupt BDNF, and tyrosine kinase B (TrkB) receptor mediated extracellular signal-regulated kinase (ERK) and thymoma viral proto-oncogene (Akt) pathways in the hippocampus and PFC [178]. Administration of antidepressants can rapidly activate TrkB, which is required for behavioral effects [179], and increase levels of ERK1 and ERK2 in the hippocampus and PFC [180, 181]. Reduction in Akt activity in ventral tegmental dopamine neurons is associated with increased susceptibility to social defeat stress, while chronic antidepressant treatment increases active Akt levels [182]. Furthermore, evidence suggests that mitogen-activated protein kinase (MAPK) modulation plays an important role in the antidepressant response. Administration of a MAPK pathway inhibitor produces depressive-like behavior and blocks effects of antidepressants in rodents [183]. Postmortem studies revealed increased expression of a negative regulator of MAPK, MAPK phosphatase-1, in the hippocampus of patients with major depressive disorder. Similar results were observed in rat and mouse models of depression, and levels could be normalized by chronic antidepressant treatment [184].

Postmortem studies on depressed suicide patients have suggested a significant reduction in mRNA and protein levels of PKA and CREB in the hippocampus and orbitofrontal cortex [185]. Overexpression of CREB in the hippocampus of rats produces an antidepressant effect in learned helplessness and forced swimming tests [186]. Chronic administration of different classes of antidepressants increases levels of cAMP production, PKA activation, and expression of CREB in the PFC and hippocampus [171, 187, 188]. In addition, CREB phosphorylation and CREB-mediated gene transcription are upregulated by chronic antidepressant treatment [180, 189]. These observations suggest an important role of the cAMP-PKA-CREB pathway in antidepressant effects.

#### 4.2.3. Glutamate Receptors (GluRs)

Stress and depression can cause dendritic remodeling and reduction in synaptic spines, while enhancement of glutamate seems crucial for these structural and functional changes [190]. GluRs are involved in modulation of neural plasticity after chronic treatment with antidepressants. Chronic administration of antidepressants fluoxetine, desipramine, and reboxetine reduces depolarization-evoked glutamate release in the hippocampus [191]. Fluoxetine increases the phosphorylation of the AMPA receptor GluR1 subunit [192] and upregulates the expression of the NMDA receptor NR2A subunit, GluR1, and GluR2 in the forebrain [159]. An AMPA receptor antagonist can reverse most antidepressant actions of fluoxetine in stressed mice [193]. A similar effect was found in the antidepressant-like effect caused by administration of lithium in the mouse tail suspension and forced swimming tests [194]. Imipramine alters ligand binding to the NMDA receptor complex in the cerebral cortex and enhances the synaptic expression of GluR1 in the hippocampus, but attenuates glutamatergic transmission and field potentials in ex vivo rat frontal cortex slices [195–198]. These data suggest an important involvement of the glutamatergic system in antidepressant action. Therefore, GluRs may represent promising targets for antidepressant development.

### 4.3. Rapid-Acting Antidepressant Ketamine

Discovery of the noncompetitive NMDA receptor antagonist, ketamine, urges us to conduct further research on the mechanisms involved in depression and to develop novel fast-acting antidepressants. Compared with classical antidepressants, ketamine exerts a robust, rapid (within a few hours), and sustained (lasts for 1 week) antidepressant effect that can be induced by a single dose in patients with treatment-resistant depression [199, 200] and in animal models of depression [177, 201, 202].

#### 4.3.1. Increased Neural Plasticity Caused by Ketamine and the Underlying Mechanism

Compared with traditional monoamine-based antidepressants, ketamine has a more direct and rapid influence on the glutamatergic system and synaptic plasticity. Ketamine rapidly reverses decreased expression of synaptic proteins and spine numbers as well as the frequency and amplitude of excitatory postsynaptic currents in PFC neurons caused by chronic stress exposure [203, 204]. Stimulus-evoked somatosensory cortical responses increase after ketamine infusion in patients with treatment-resistant depression, which suggests increased cortical excitability [205, 206].

Antidepressant effects of ketamine might be related to enhanced expression of AMPA receptors and BDNF [207, 208]. It was reported that ketamine reduced phosphorylation of eukaryotic elongation factor 2 kinase and disinhibited translation of BDNF [202]. However, another study showed that ketamine produced similar antidepressant-like responses in wildtype and heterozygous BDNF knockout mice, and it did not influence levels of BDNF or TrkB phosphorylation in the hippocampus [177]. The mammalian target of rapamycin (mTOR) pathway, as a downstream signaling cascade of BDNF, has been implicated in protein synthesis-dependent synaptic plasticity and can be interrupted in depression. Compared with healthy controls, expression levels of mTOR and its core downstream signaling target proteins, p70S6K, elF4B, and p-elF4b, are reduced significantly in depressed individuals [209]. Levels of regulated in development and DNA damage responses-1, an inhibitor of mTOR, increase in the PFC of patients with depression, along with a concurrent decrease in phosphorylation of signaling targets of mTOR [210]. Ketamine can activate the mTOR pathway, which leads to an increase in synaptic signaling proteins and new spine synapses. Blockade of mTOR signaling can completely block ketamine-induced synaptogenesis and behavioral responses in models of depression [203].

As a key component of the Wnt pathway and upstream of the mTOR signaling cascade, glycogen synthase kinase 3-*β* (GSK3-*β*) plays major roles in gene expression, cell behaviors, neurodevelopment, and regulation of neuronal plasticity [211]. It contributes to synaptic deconsolidation and shows increased levels in brains of patients with major depressive disorder [212]. A promoter single nucleotide polymorphism of GSK3-*β* (rs334558) is associated with delayed onset of depression [213] and an improved response to lithium salt therapy [96]. Antidepressant effects of ketamine require an inhibitory phosphorylation of glycogen synthase kinase-3 (GSK3) and can be potentiated when administered with the nonselective GSK3 inhibitor lithium chloride [154, 214].

#### 4.3.2. SWA Changes as a Predicator of Ketamine-Induced Plasticity and Antidepressant Effects

SWA is considered a sensitive marker of cortical synaptic strength and synchronization [215–217]. In patients with depression, SWA and delta sleep ratio (DSR, the ratio of SWA between the first 2 NREM sleep episodes) tend to be lower [83, 218]. Reduction in delta power during NREM sleep is linearly associated with improved negative affect in major depressive disorder [219]. The measure of distribution of SWA and DSR might be a more robust predictor of clinical response and recurrence to antidepressant therapy than REM sleep latency. A higher DSR may indicate more favorable therapeutic outcomes [83, 218]. Similar to some conventional antidepressants [218, 220], administration of ketamine increases SWA and DSR in rats [221] and individuals with depression [222, 223]. It is noteworthy that the decrease in plasma BDNF levels of depressed patients is proportional to the change in EEG parameters [223]. These studies suggest a role of SWA and DSR in predicating ketamine-induced neural plasticity changes and antidepressant effects.

## 5. Neural Plasticity Involved in Antidepressant Effects of Therapeutic Sleep Deprivation (SD)

### 5.1. SD Therapy for Depression

Since it was first found to benefit depressed patients in the 1970s, therapeutic SD has been widely used as a rapid antidepressant treatment. SD shows a rapid and robust antidepressant effect in patients with broadly defined depression, including some difficult-to-treat conditions [224, 225]. The effect of therapeutic SD is highly reproducible and substantial, but transient. Most patients relapse after 1 night of sleep or even short naps [225, 226], which limits SD as the first-line treatment for depression. Some new clinical strategies have been developed to sustain the efficacy of SD, including combining SD with chronobiological techniques (light therapy and sleep-phase advance) or antidepressants [227–229].

### 5.2. SD and Neural Plasticity

#### 5.2.1. Changed Sleep Homeostasis and Neural Plasticity

Similar to other rapid-acting antidepressant treatments such as rapid-acting NMDA receptor antagonist or electroconvulsive therapy, SD regulates neuronal inhibition-excitation balance in the brain. Nocturnal sleep following SD in patients who respond positively to SD therapy show a higher rebound of sleep wave sleep (SWS) compared with those that respond negatively [88]. Studies have suggested that changes in SWA may be associated with the therapeutic outcome of SD, and a high baseline DSR is a positive predictor for SD response [230]. A SWS deprivation test proved that a reduction in depressive symptoms was correlated with overnight dissipation of frontocentral SWA on baseline sleep, rebound in right frontal all-night SWA on recovery sleep, and amount of REM sleep on the deprivation night [231]. These data indicate a change in sleep homeostasis of depressed patients during SD therapy.

Neuroplasticity also contributes to antidepressant effects of therapeutic SD. SD was reported to increase dendritic spine density in the dentate gyrus of the hippocampus, which was associated with upregulation of Wnt signaling gene Wnt 7a and activation of the innate immune system of the brain. Increased expression of the immediate early Arc/Arg3.1 suggests an increased neuroplasticity [232]. In addition, similar to the rapid-acting NMDA receptor antagonist ketamine, an increase in inhibitory phosphorylation of the signaling protein GSK3-*β* contributes to the antidepressant effect and synaptic potentiation of therapeutic SD [67]. Its single nucleotide polymorphism, rs334558, influenced acute antidepressant response of SD and showed a better mood elevation [233]. A role for glutamatergic neurotransmission has also been reported. A molecular imaging study demonstrated that therapeutic SD induced an increase of cerebral functional mGluR5 availability, which is consistent with reduced density of mGluR5 in depressed patients [234]. Moreover, increased cortical plasticity, indicated by increasing cortical excitability, was reported during repeated SD in patients with bipolar disorder, which paralleled and predicted the antidepressant response to SD. This may be a major effect of successful antidepressant treatments, and patients who do not respond may experience persistent impairment in neuroplasticity mechanisms [235].

#### 5.2.2. A Synaptic Plasticity Model of SD in Depression

According to the classic 2-process model of sleep regulation, depression develops because of a deficient build-up of homeostatic process S with an unaffected circadian process C. Therapeutic SD benefits from a transient increase of process S [236]. When linked to the recent SHY where synaptic strength changes during the sleep/wake cycle, the therapeutic effect of SD is likely due to changed synaptic potentiation [65, 71].

A rat study showed that electrically induced LTP was occluded partially during prolonged SD and restored after sleep [237]. However, prolonged wakefulness beyond a physiological duration did not further increase spine density [69]. Therefore, Wolf et al. [238] concluded that SD might lead to excessively high cortical excitability and saturation of synaptic strength and, consequently, to partial occlusion of LTP inducibility. They further postulated a window of optimal associative synaptic plasticity (LTP inducibility) during wakefulness. After sleep (insufficient upscaling) and extended periods of sleep deprivation (saturation), LTP inducibility is reduced. Based on this, a synaptic model was proposed. It was hypothesized that in patients with depression, LTP inducibility is impaired and the window of optimal associative plasticity may not extend through a normal waking period because the ability to generate cortical LTP diminishes. Therapeutic SD enhances cortical synaptic strength and therefore shifts deficient LTP inducibility in depressed patients to a more favorable window of associative plasticity. Namely, in healthy controls, SD leads to synaptic saturation and deficient LTP inducibility, but it compensates for attenuated synaptic plasticity in the brains of patients with depression and finally evokes an antidepressant effect. The model builds on changed synaptic strength and cortical excitability in healthy people and depressed patients during different stages of wake/sleep cycles. It explains the paradoxical role of SD in dampening neural plasticity in healthy controls and improving clinical symptoms in patients with depression. Further research must be done to evaluate the validity of this model.

## 6. Conclusion

In this review, we summarize the latest progress on the mechanisms of interactions between sleep, depression, and neural plasticity. Although there have been much excitements with recent progress in sleep-related methods to treat depression via regulation of neural plasticity, further development and clinical application are needed to elucidate the mechanisms and their effects.
